# Comparison of the diagnostic accuracy of the Pluslife Mini Dock RHAM technology with Abbott ID Now and Cepheid GenXpert: A retrospective evaluation study

**DOI:** 10.1038/s41598-024-64406-9

**Published:** 2024-06-17

**Authors:** Laura Herrmann, Juliana Breuer, Tuan Ngo Duc, Nicole Thomé, Fatemeh Ghazaani, Sundrela Kamhieh-Milz, Julian Kamhieh-Milz, Andreas Pfützner

**Affiliations:** 1 DHS – Diagnostic HealthCare Solutions, Berlin, Germany; 2 Lifecare Laboratories, Mainz, Germany; 3 Pfützner Science and Health Institute, Mainz, Germany; 4 Institute of Transfusion Medicine, Charité – Universitätsmedizin Berlin, Robert-Koch Platz 4, 10117 Berlin, Germany; 5 Institute for Internal Medicine and Laboratory Medicine, University for Digital Technologies in Medicine and Dentistry, Wiltz, Luxembourg

**Keywords:** Assay systems, Infectious-disease diagnostics, Virology

## Abstract

Rapid and sensitive detection of pathogens is critical in interrupting the transmission chain of infectious diseases. Currently, real-time (RT-)PCR represents the gold standard for the detection of SARS-CoV-2. RNase HII-assisted amplification (RHAM) is a promising technology, enabling reliable point-of-care (PoC) testing; however, its diagnostic accuracy has not yet been investigated. The present study compared the Pluslife Mini Dock (RHAM technology), with Abbott ID Now and Cepheid GeneXpert IV. The positive percent agreement (PPA) and negative percent agreement (NPA) were determined in 100 SARS-CoV-2 positive and 210 SARS-CoV-2 negative samples. Further, the reliability of the Pluslife Mini Dock was investigated in different SARS-CoV-2 variants (Delta and Omicron subvariants). The PPA was 99.00% for Pluslife, 100.00% for Abbott ID Now, and 99.00% for Cepheid GeneXpert, with an NPA of 100.00%, 98.90%, and 93.72%, respectively. Abbott ID Now demonstrated the highest rate of invalid results. All SARS-CoV-2 analysed variants were detected by the Pluslife device. Altogether, the Pluslife Mini Dock demonstrated a PPA of 99.16% (235/237) for C_T_ < 36 and an NPA of 100.00% (313/313), respectively. In conclusion, the Pluslife Mini Dock demonstrated better analytical performance than Abbott ID Now and Cepheid GeneXpert IV, representing a highly accurate and rapid PoC alternative to RT-PCR.

## Introduction

Since the initial outbreak of severe acute respiratory syndrome coronavirus 2 (SARS-CoV-2) in December 2019, the virus has spread rapidly, resulting in a global pandemic^1,2^. The highly transmissible RNA virus causes a respiratory disease termed Coronavirus disease 2019 (COVID-19), with a clinical spectrum ranging from asymptomatic and mild symptoms to severe respiratory infection, pneumonia, and even death^1,3,4^. As of August 2023, more than 765 million confirmed cases of COVID-19 and almost 7 million deaths have been reported to the World Health Organization (WHO)^5^.

In May 2023, WHO declared an end to the COVID-19 global emergency^5^. In retrospect, there are numerous lessons that have been learned from this recent pandemic. Undoubtedly, rapid antigen tests (RATs) were a game changer in fighting the spread of infections due to early detection. However, RATs demonstrate reduced sensitivity and are often unable to detect the pathogen early after exposure, particularly in the absence of symptoms. Further, rapid testing devices are most accurate in people with signs or symptoms of COVID-19. Real-time PCR (RT-PCR) has emerged as the gold standard for the detection of SARS-CoV-2 infection, with high precision and sensitivity^6,7^. However, during the pandemic’s early stages, laboratories worldwide quickly reached their limits in terms of testing capacity. Consequently, results were often available several days after sampling. Additionally, RT-PCR requires special laboratory infrastructural conditions and facilities, cost-intensive equipment, and specially trained personnel, which may not be available in some regions^8^. Accordingly, a simple and rapid assay with comparable test accuracy to RT-PCR would more effectively help to manage any future pandemics.

Various studies have demonstrated loop-mediated isothermal amplification (LAMP) to be a promising alternative method to classical RT-PCR. The LAMP technology enables efficient and specific amplification of DNA and RNA (RT-LAMP) without an additional nucleic acid extraction under isothermal conditions in under 60 min^9,10^. LAMP-based assays have been developed successfully for the diagnosis of several disease-causing infectious agents, such as *Plasmodium spp.* (malaria)^11,12^, Zika virus^13^, *Mycobacterium tuberculosis*^14,15^, and SARS-CoV-2^16–18^. However, non-specific amplification remains a problem in many LAMP-based assays, and the false negative rate compared to RT-PCR is significantly higher^17^. A new isothermal amplification method termed RHAM (RNase HII-assisted Amplification) was described recently, as an alternative rapid and easily adaptable molecular diagnostic platform. It is comprised of LAMP-mediated exponential amplification, employing an RNase HII reporter for signal visualisation in a single reaction^19^. RHAM can detect positive samples in as little as 20 min without the need for a laboratory-based environment and facilities. However, the test accuracy of this technology in clinical samples has not yet been determined.

The present study describes the first independent evaluation study of the RHAM technology using SARS-CoV-2 as a model pathogen. To better understand the differences between RHAM and other PoC testing devices (POCT), the RHAM-technology based Pluslife Mini Dock test was compared to Abbott ID Now™ (NEAR technology) and Cepheid GeneXpert® (RT-PCR). Additionally, the clinical accuracy of the RHAM technology towards the accurate detection of SARS-CoV-2 subvariants was investigated.

## Methods

### Study sites

This retrospective study involved two independent study sites. A comparative study using the Pluslife Mini Dock (Guangzhou Pluslife Biotech, Guangzhou, China), Abbott ID Now™ (Abbott Diagnostics, Wiesbaden, Germany), and Cepheid GeneXpert® IV (Cepheid, Krefeld, Germany) was conducted at the Pfützner Science & Health Institute in co-operation with the Institute of Internal Medicine and Laboratory Medicine of the University for Digital Technologies in Medicine and Dentistry, Wiltz, Luxembourg (Mainz, Germany; study site 1). The second study site was DHS—Diagnostic HealthCare Solutions GmbH, Berlin, Germany, in co-operation with the Institute of Transfusion Medicine, Charité Universitätsmedizin Berlin, Germany.

### Study design and clinical samples

For the comparative study at study site 1, 319 residual nasopharyngeal swab samples (100 RT-PCR positive samples with C_T_ < 36, nine samples with a C_T_ of > 36, and 210 RT-PCR negative samples) were analysed. Samples had been stored in 1 mL Universal Transport Medium (Copan UTM™, Brescia, Italy) at − 80 °C for less than 6 months. The swab samples used in this study were residual samples (stored at − 80 °C) collected during a previous clinical performance evaluation study (NAL-CoV-002), which had been approved by the responsible IRB (Ethikkommission der Landesärztekammer Rheinland-Pfalz—Application No. 2022–16,548). All participating subjects had signed a written informed consent for this study (also approved by the IRB), where they explicitly approved the storage of their residual sample material and future use for laboratory evaluation projects in an anonymous fashion.

At study site 2, 137 SARS-CoV-2 RT-PCR confirmed positive and 104 negative samples were analysed. All positive samples were previously subtyped for the major SARS-CoV-2 variants. Samples included in the study were residual samples from diagnostic testing that were used in anonymised form and stored for a maximum of 2.5 years at − 80 °C. Clinical remnant samples do not require informed consent from the subject regulating the further use of the specimens.

As residual samples in anonymised form were used, ethics approval was not required. All methods were performed in accordance with relevant guidelines and regulations.

PBS (Carl Roth, Karlsruhe Germany) was used as a transport medium for nasopharyngeal and/or oropharyngeal swabs at study site 2. Samples of the Delta variant and several BA.1 (8/20) subtyped samples were stored in virus-inactivating transport medium (VTM; Biocomma, Guangdong, China). Unfortunately, VTM is incompatible with the enzyme used by Pluslife in the RHAM technology (data not shown). Accordingly, 10 µL of eluates were added to 490 µL of the lysis buffer provided by the Pluslife test kits.

### Viral RNA extraction

For RNA extraction, the QuickExtract RNA extraction kit (Lucigen, Wisconsin, USA) using a one-tube approach was applied by study site 1. At study site 2, RNA extraction was performed using the NEOS-32 XT extractor (Linear, Barcelona, Spain) in combination with the NukEx Mag RNA/DNA extraction kit (Gerbion, Kornwestheim, Germany). All extractions and PCRs were performed according to the manufacturer´s instructions.

### Real-time PCR

Study site 1 performed RT-PCR using the EURO RealTime SARS-CoV-2 PCR kit (Euroimmun Medizinische Labordiagnostika AG, Luebeck, Germany) on the Quant Studio 5 (Thermo Fisher, Waltham, Massachusetts, USA). A C_T_ < 36 of the combined target genes ORF1ab and N was determined as SARS-CoV-2 positive. Study site 2 performed RT-PCR using the virellaSARS-CoV-2 s 2.0 real time PCR kit (Gerbion) for detection of the RdRp + S and E genes or the respiraScreen 1 RT PCR kit (Gerbion) for the simultaneous detection of SARS-CoV-2 (RdRp + E), Influenza A/B, and respiratory syncytial virus (RSV). PCR was performed using the LightCycler® 480 II (Roche, Mannheim, Germany) or MIC-IVD (BMS—Bio Molecular Systems, Upper Coomera, Queensland, Australia).

### *SARS-CoV-2 subvariant analysis *via* RT-PCR*

SARS-CoV-2 subvariant determination was performed using the GenXPro VoXcreen assays (GenXPro GmbH, Frankfurt am Main, Germany) for Delta/Omicron, Omicron BA.1/2, Omicron BA.2/4/5, or Omicron/XBB differentiation, and/or the virellaSARS-CoV-2 Mutant 3 real time PCR kit. PCR was performed using the Roche LightCycler® 480 II or MIC-IVD.

### Comparative analysis of PoC devices and tests for SARS-CoV-2

The following PoC devices were compared: Pluslife Mini Dock using the SARS-CoV-2 Nucleic Acid Testing Card (both from Guangzhou Pluslife Biotech), Abbott ID NOW™ system using the ID NOW™ COVID-19 test (both from Abbott), and Cepheid GeneXpert® IV using the Xpert® Xpress SARS-CoV-2 test kit (both from Cepheid). All PoC testing were performed according to the manufacturer´s instructions, with the exception of the sample material. All manufacturers describe the addition of the collected nasopharyngeal swab to the corresponding supplied lysis buffer. In the present study, swabs were dissolved initially in UTM or PBS, which was added subsequently to the lysis buffer of the test kit. For the Xpert® Xpress SARS-CoV-2 assay, 300 µL UTM was used. In contrast, for the two remaining assays, 200 µL UTM was used per sample and added to the respective lysis buffer provided.

At study site 2, 250 µL of the PBS sample supernatant was mixed with 250 µL of lysis buffer, which was subsequently added in the appropriate volume to the test cassette (~ 440 µL). As described above, extracted nucleic acid was used in some cases, with samples diluted 1:50.

### Statistical analysis

The positive percent agreement (PPA), negative percent agreement (NPA), positive and negative predictive values (PPV and NPV, respectively) including 95% confidence intervals of the PoC tests were calculated in comparison to the reference method (RT-PCR) using an open-source online tool: https://www.medcalc.org/calc/diagnostic_test.php. To assess the agreement between the reference PCR and the PoC test results, the following parameters were calculated: Positive precent agreement (PPA, sensitivity) was calculated by evaluating the number of positive PCR samples, which are correctly detected as “positive” also by the study test. Negative percent agreement (NPA, specificity) was calculated by evaluating the number of negative PCR samples, which are correctly detected as “negative” by the study test. The total accuracy was calculated by determining the number of samples where both tests (PoC test and reference method) demonstrated valid results. The overall agreement demonstrates correct results by the POC tests in relation to the total amount of samples tested. Statistical analysis and scientific plots were performed using GraphPad Prism V9 for Windows, (GraphPad Software, Boston, Massachusetts USA). Populations were tested for normal distribution using the D´Agostino-Pearson omnibus normality test. Variant analysis was performed for non-parametric cohorts using the Kruskal–Wallis test. Pearson correlation was calculated for populations that were normally distributed. A *P* value < 0.05 was considered statistically significant.

## Results

### Comparison of the PPA, NPA, and accuracy of the three analysed PoC devices

The PPA, NPA, and accuracy were determined for all three POCTs (Table 1). Identical samples were used in a paired approach. Overall, 319 samples were investigated. Among the 100 RT-PCR confirmed positive samples, the Pluslife Mini Dock demonstrated a PPA of 99.00% (95% CI, 94.55–99.97%). There was one invalid result using the Abbott PoC device (ID 018 with a C_T_ of 33.27). All other valid positive samples (n = 99) were correctly detected as positive, revealing a PPA of 100.00% (95% CI, 96.34–100.00%). There was one invalid sample using the Cepheid PoC device in addition to one false negative result (ID 018), revealing a PPA of 98.99% (95% CI, 94.50–99.97%). The positive predictive value (PPV) was 100.00% (95% CI, 96.34–100.00%) for Pluslife, 98.02% (95% CI, 92.58–99.49%) for Abbott ID Now™, and 88.29% (95% CI, 81.64–92.74%) for Cepheid GeneXpert®.

**Table 1:** Comparison of sensitivity, specificity and accuracy of Pluslife Mini Dock SARS-CoV-2, Abbott ID NOW™ COVID-19, and Cepheid Xpert® Xpress SARS-CoV-2.

		Pluslife	Abbott	Cepheid
Sensitivity	Number of invalid tests	0	1	1
	C_T_ < 25 (n = 39)	39/39	39/39	38/38
	C_T_ < 30 (n = 75)	75/75	75/75	74/74
	C_T_ < 36 (n = 100)	99/100	99/99	98/99
	Overall sensitivity	99.00%	100.00%	98.99%
Specificity	Number of invalid tests	1	30	3
	Confirmed negative findings	209/209	178/180	194/207
	Overall specificity	100.00%	98.90%	93.72%
Positive predictive value (PPV)		100.00%	98.02%	88.29%
Negative predictive value (NPV)		99.52%	100.00%	99.52%
Positive predictive agreement (PPA)		99.00%	100.00%	98.99%
Negative predictive agreement (NPA)		100.00%	98.90%	94.09%
Total accuracy		99.68% (308/309)	99.29% (277/279)	95.42% (292/306)
Overall agreement		99.35% (308/310)	89.35% (277/310)	94.19% (292/310)

Of the 210 RT-PCR confirmed negative samples, the Pluslife Mini Dock demonstrated one invalid result but confirmed all 209 samples as negative (100% NPA). Abbott ID Now™ demonstrated invalid results for 30 samples. From the remaining 180 samples, there were two false positive results, revealing an NPA of 98.90%. In contrast, there were three invalid results using the Cepheid GeneXpert® IV. From the remaining 207 negative samples, 13 samples were false positive, demonstrating an NPA of 94.09%. The negative predictive value (NPV) was 99.52% (95% CI, 96.75–99.93%) for Pluslife, 100% (95% CI, 97.97–100.00%) for Abbott ID Now™, and 99.52% (95% CI, 96.71–99.93%) for Cepheid GeneXpert®.

The overall accuracy was found to be 99.68% for Pluslife (95% CI, 98.21–99.99%), 99.29% for Abbott (95% CI, 97.45–99.91%), and 95.42% for Cepheid (95% CI, 92.75–97.58%). Among all 310 samples, evaluable and correct results (overall agreement) were found in 99.35% of samples by Pluslife (308/310), 89.35% by Abbott (277/310), and 94.19% by Cepheid (292/310). Overall, with 31 samples (both positive and negative), the highest number of invalid results were observed with the Abbott ID Now™, followed by Cepheid GeneXpert® IV with four samples, and the Pluslife Mini Dock with only one sample.

### ***Limitation of POCT for samples with a low viral load (C***_***T***_ > ***36)***

Samples with a C_T_ value < 36 were termed as positive, whereas those with a C_T_ value > 36 were termed as negative; however, the results were considered as representing a “grey zone” for the POC test evaluation. Accordingly, nine samples with a C_T_ > 36 (36.27–37.73) were excluded from statistical analysis, as it was not possible to definitively conclude whether these samples were negative or positive for SARS-CoV-2.

In the direct comparison of this sample subset, the Pluslife Mini Dock demonstrated valid negative results in all nine cases. Abbott ID Now™ demonstrated one invalid, two positive, and six negative results. Using the Cepheid GeneXpert® IV, three SARS-CoV-2 positive results and six negative results were determined.

### Clinical accuracy in the identification of SARS-CoV-2 variants using the Pluslife Mini Dock POC device

**Figure 1:**
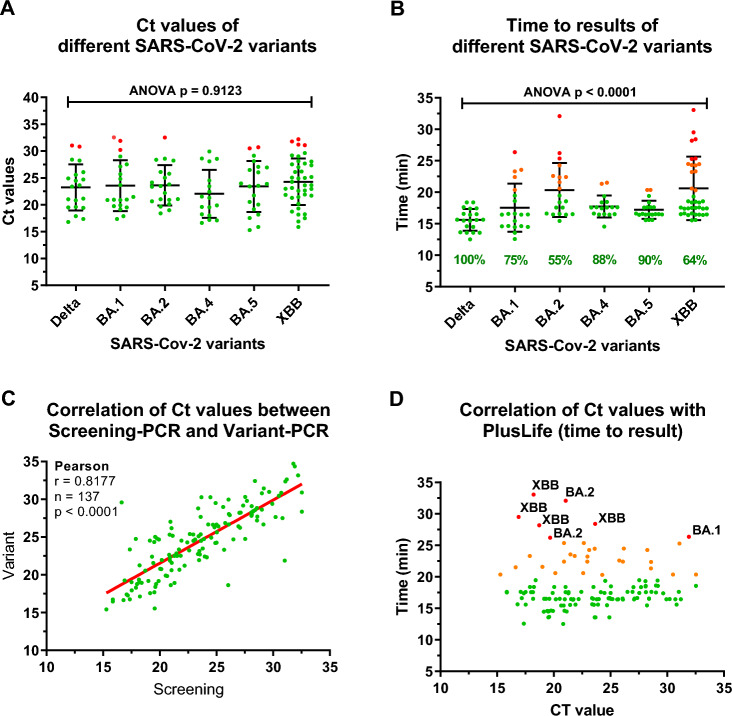
Clinical accuracy of the identification of SARS-CoV-2 subvariants using the Pluslife Mini Dock based on the RHAM technology. (**A**) Distribution of C_T_ values across SARS-CoV-2 variants. There were no significant differences in C_T_ values, which ranged from 15.29–32.53 (mean, 23.5 ± 4.5; One-Way ANOVA, *P* = 0.9123). (**B**) Time-to-results across the SARS-CoV-2 variants. Dilutions of 1:50 of the eluates of the Delta (100%) and BA.1 (50%) typed samples were performed as virus-inactivating transport medium is not compatible with the enzyme reaction present in the Pluslife test card. Accordingly, RNA eluates were detected earlier. Samples of the BA.2 to XBB variants were PBS supernatants. Compared to the other variants, samples of the XBB variant had a time-to-result (> 20 min). (**C**) Correlation between C_T_ values obtained by the SARS-CoV-2 screening PCR and different PCR-based variant analysis kits used. Overall, C_T_ values were highly reproducible (Pearson r = 0.8177, *P* < 0.0001, n = 137). (**D**) Correlation of C_T_ values with the time-to-positive result. There was no correlation between the virus load and the time-to-positive results (Pearson r = 0.014, *P* = 0.8695, n = 135). In conclusion, 76.3% of the samples were positive within 20 min, 92.6% of the samples were positive within 25 min, and only 7.4% were positive after more than 25 min (max. 32 min). Two samples with a relatively low viral load (C_T_ of 31.79 and 32.20) demonstrated negative results.

The C_T_ values of the SARS-CoV-2 positive samples of the variants Delta (n = 20), BA.1 (n = 20), BA.2 (n = 20), BA.4 (n = 17), BA.5 (n = 20), and XBB (n = 40) are depicted in Fig. 1. With the exception of one XBB sample that was found to be false negative (C_T_ > 31.79), all remaining 136 analysed samples were detected as positive. The PPA was 99.27% (95% CI, 96.00–99.98%) and the NPA 100% (104/104; 95% CI, 96.52–100.00%). With an accuracy of 99.59% (95% CI, 97.71–99.99%), the results of study site 1 were reproduced and confirmed by study site 2.

### Impact of SARS-CoV-2 variants on time-to-result

The mean C_T_ values and time-to-results for the analysed SARS-CoV-2 variants are depicted in Table 2. There was no significant difference in regard to the C_T_ values between the various SARS-CoV-2 variants (One-way ANOVA, *P* > 0.05). However, there was a significant variation in the time-to-positive results across the different SARS-CoV-2 variants (*P* < 0.0001).

**Table 2:** SARS-CoV-2 variants and impact on time-to-result.

SARS-CoV-2 variant	Mean ± SD	Time (Min–Max) (min)	Sample number
Delta	15.62 ± 1.73	12.52–18.38	n = 20
BA.1	17.54 ± 3.83	12.57–26.50	n = 20
BA.2	20.35 ± 4.28	15.43–32.05	n = 20
BA.4	17.74 ± 1.75	14.53–21.52	n = 17
BA.5	17.21 ± 1.44	15.57–20.35	n = 20
XBB	20.62 ± 5.05	15.53–35.10	n = 39

In the primary setting, 20 samples of the XBB variant were initially analysed. The mean test duration of 22.89 ± 5.67 min for XBB was significantly higher compared to all other variants, which demonstrated a mean of only 17.69 ± 3.23 min (n = 97) (*p* < 0.0001). Therefore, we analysed an additional 20 samples of the XBB variant to exclude a statistical variance. All additional samples were confirmed as positive, with a mean time-to-result of 18.44 ± 2.89 min that demonstrate no statistically significant difference to the other variants (*p* = 0.3411). The mean time-of-duration for all 39 XBB positive samples was now found to be 20.66 ± 4.98 min, which is more comparable with the other variants analysed. Renewed statistical analysis with the increased number of SARS-CoV-2 positive samples of the XBB variant showed that the mean test duration was still significantly higher compared to some other variants (Mann Whitney U test, Delta *P* < 0.0001, BA.1 *P* = 0.0055, BA.2 *P* = 0.9210, BA.4 *P* = 0.1566, BA.5 *P* = 0.0083).

### Declaration of specific target amplification and cross-reactivity

A longer measuring time may represent a sign of suboptimal target amplification efficiency. Therefore, we consulted Pluslife regarding the potential impact of mutations on their isothermal amplification efficiency. They declared no false negative results for the following variants: Alpha (B.1.1.7/Q7), Beta (B.1.351/B1.351.2/B1.351.3), Gamma (P.1/P.1.1/P.1.2), Delta (B1.617.2/AY.1/AY.2/AY.3/AY.3.1/AY.4/AY.4.2/AY.5/AY.5.1/AY.5.2/AY.6 AY.7/AY.8/AY.9/AY.10/AY.25/AY.27/AY.30/AY.70/AY.74/AY.88/AY.107) and Omicron (B.1.1.529/BA.1/BA.1.1/BA.2 /BA.2.2/BA.2.75/BA.2.75.2/BA.2.12/BA.2.12.1/BA.3/BA.4/BA.5/BA.52/XE/BQ1.1/BQ1/XBB.1.5 and the WHO variants of interest labelled variants Eta (B.1.525), Theta (P.3) Iota-(B.1.526), Kappa (B.1.617.1), Lambda (C.37), Mu (B.1.621/B.1.621.1), and the variants Epsilon (B.1.427/B.1.429), Zeta (P.2) XD, and XF.

Additionally, the PoC test cards from Pluslife used in the present study were recently reported to demonstrate no cross-reactivity with synthesised nucleic acids of other respiratory pathogens, such as coronavirus (HKU1, OC43, NL63, 229E), SARS coronavirus (SARS-1), MERS coronavirus, influenza A virus (H1N1, H5N1, H7N9 and H9N2), influenza B virus, respiratory syncytial virus types A and B, human parainfluenza virus type II, adenovirus types 3 and 7, enterovirus EV71, *Mycoplasma pneumoniae*, EB virus, human cytomegalovirus, and *Mycobacterium tuberculosis*^19,20^.

### ***Correlation of time-to-result with C***_***T***_*** values***

Compared to RT-PCR, POCT usually do not usually provide C_T_ values, which also applies to the RHAM technology applied in this study. As the reaction stops when the fluorescence signal reaches the threshold, we assumed that the duration of the reaction may correlate with the C_T_ value of a positive sample.

A significant correlation can be observed between the C_T_ values obtained by the screening PCR primarily using the Gerbion virellaSARS-CoV-2 PCR kit and those of the GenXpro variant kits (Fig. 1c; Pearson r = 0.8177, *P* < 0.0001, n = 137). This correlation also represents sample stability and reproducibility. However, there was no correlation between C_T_ value and time-to-positive results (Pearson r = 0.01427, *P* = 0.8695). This implies that, despite C_T_ values spanning from 15 to 32, positive results were obtained within 20 min (76.3%) in the majority of cases. In 92.6% of the samples, a positive test result was obtained within 25 min.

## Discussion

Due to its high sensitivity and specificity, RT-PCR is considered the gold standard for the detection of SARS-CoV-2^6,7^. However, to maintain these very good performance qualities, this technology requires a sound laboratory infrastructure, trained professionals, and expensive equipment and facilities, which limits the accessibility to PCR testing, particularly in developing countries^8,19^. From this aspect, LAMP appears to be a promising alternative to PCR, as it can be implemented as a PoC method, with equipment costs much lower compared to PCR^8,10,17,20^. However, an improvement of the diagnostic sensitivity and specificity is required to be comparable with RT-PCR.

In an emergency diagnostic situation, as observed in the recent SARS-CoV-2 pandemic, a short sample-to-result time is essential for successful epidemiological disease management. As RT-PCR approaches usually take between 3–6 h for analysis (excluding transport time, pre-analytics, and reporting), one of the main advantages of POCT is that results are provided within minutes compared to several hours or days (see Table 3).

**Table 3:** Characteristics of the Pluslife Mini Dock, Abbott ID Now, and Cepheid Xpert Xpress POC devices.

	Pluslife Mini Dock SARS-CoV-2	Abbott ID NOW™ COVID-19	Cepheid Xpert® Xpress SARS-CoV-2
Manufacturer	Guangzhou Pluslife Biotech	Abbott Diagnostics	Cepheid
POCT device	Mini Dock	ID Now	GeneXpert IV
Samples per run	1–8^+^	1	1
Cost per device	< 1000 €	~ 2500–3500 €	~ 4500 €
Cost per Test	9.90 €	31.60 €	Unknown
Technology	LAMP/RHAM	NEAR	RT-PCR
Time-to-result*	7–20 min (pos) to 35 min (neg)	6 (pos) to 13 min (neg)	20–25 min
Dimensions: W|L|H (cm)	8.6|10.0|6.3	20.7|14.5|19.4	27.9|30.5|29.7
Handling time	1–2 min	Unknown	~ 1 min
SARS-CoV-2 target sequences	N and ORF1ab	RdRp	E and N2

*LAMP* Loop-mediated isothermal amplification, *NEAR* Nicking enzyme amplification rection; *neg.* negative, *POCT* Point-of-care testing; pos., positive, *RHAM* RNase HII-assisted amplification.

*Information is in accordance with the manufacturer.

^+^One using a single Mini Dock, five samples using the 5-port hub, and eight samples using the Pro Dock PCE device.

Although isothermal nucleic acid amplification and LAMP are comparable to RAT in terms of simplicity, convenience, and rapidity, their sensitivity and specificity are far more superior^19,21,22^. Based on our experience and according to various studies, false negative RAT results have been observed in samples with a low viral load, particularly in samples with a C_T_ > 25^23–25^. In a pandemic setting, this may represent a burden as contact persons desire rapid results to exclude potential infection. The application of RATs at a very early stage after contact may reveal false negative results. Therefore, the is a high demand for more sensitive rapid diagnostic tests. A relatively high number of different isothermal nucleic acid amplification techniques have been developed and described, including but not limited to exponential amplification reactions (EXPAR), exponential rolling circle amplification (E-RCA), exponential strand displacement amplification (E-SDA), helicase-dependent amplification (HDA), nucleic acid sequence-based amplification (NASBA), and recombinase polymerase amplification (RPA)^9,26–30^. However, these techniques still have several shortcomings, with non-specific amplifications representing the largest hindrance for their rapid implementation in routine use as discussed elsewhere^19,31,32^. New LAMP-PoC devices are under continuous development and require clinical evaluation. The diagnostic accuracy of the recently described RHAM technology^19^, which uses an RNase HII reporter for signal visualisation, had not yet been investigated.

The present study is the first to report on the diagnostic sensitivity and accuracy of the RHAM technology in a PoC device (Pluslife Mini Dock) in a direct comparison to two well-established POC platforms (Abbott ID Now™ and Cepheid GeneXpert® IV), and additional performance characteristics of the Pluslife Mini Dock for the detection of SARS-CoV-2 variants. For this study, previously collected nasopharyngeal and oropharyngeal swab samples stored in UTM or PBS at -80 °C were used, which is, however, not in accordance with the original instructions for sample collection by the manufacturers for all three tests. The foreseen sample for these kits is freshly collected dry swab samples. For example, the instructions for Abbott ID Now™ COVID-19 state “direct test performance without elution into a VTM”, whereas the Cepheid Xpert® Xpress SARS-CoV-2 assay also accepts samples stored in VTM (3 mL, Copan or similar) for a maximum 8 h at RT or a maximum of 7 days at 2–8 °C in NaCl solutions. Yet, 98.7% of the samples used in the comparative accuracy study (study site 1) demonstrated valid results. This enables the acquisition of information regarding their diagnostic accuracy. With only one invalid result, the Pluslife Mini Dock was observed to be most stable in terms of specimen variability, with 99.7% of samples examined at study site 1 demonstrating valid test results. In contrast, Abbott ID Now™ showed a total of 31 invalid results, whereas Cepheid Xpert® Xpress had four invalid results. It cannot be excluded that the use of VTM may lead to reduced test accuracy in Abbott ID Now™. However, a previously published comparison between the collection method and sample type using Abbott ID Now™ COVID-19 and Cepheid Xpert® Xpress showed that approximately one-third of the samples that tested positive by the Cepheid Xpert® Xpress were negative using Abbott ID Now™ when using nasopharyngeal swabs in VTM compared to 45% when using dry nasal swabs^33^. This may indicate that Abbott ID Now™ is more prone to invalid results, regardless of the sample type used. The use of retrospective samples in this evaluation study, however, represents a limitation.

This may also be represented by the fact that all three PoC devices applied in this study demonstrate a very good diagnostic PPA of ≥ 99.00%. Interestingly, sample ID 018 (C_T_ of 33.27) demonstrated ambiguous results upon measurement in all three devices. False negative results were observed with this sample using the Pluslife and Cepheid devices and an invalid result was observed with Abbott ID Now™, suggesting the presence of interfering substances in this particular sample. Of note, the sample was not excluded due to the uncertainty of this discrepancy and was retained in statistical analyses.

Despite the observation of 10% invalid results with Abbott ID Now™ (primarily in the negative cohort), the determined PPA of 100% is not in accordance to the literature. Sensitivities of 89.9% or even 79% (in comparison to 99% for Cepheid GeneXpert) were reported elsewhere^34,35^. However, different comparative studies regarding the PoC detection of SARS-CoV-2 show highly variable results. The PPA for Abbott ID Now™ range between 48 and 96% and for Cepheid GeneXpert® between 95 and 100%, with the studies demonstrating variable study designs and sample types^20,35^. In the present study, the PPA for the Pluslife Mini Dock was found to be 99.00%, for Abbott ID Now™ 100.00%, and for Cepheid GeneXpert® 98.99%, with a NPA of 100.00%, 98.90% and 94.09%, respectively.

Notable differences in the NPA were observed between the three PoC devices examined. Using 219 confirmed RT-PCR negative samples, the NPA was 100.00% for Pluslife. Abbott ID Now™ demonstrated an NPA of 98.90%, with two false positive cases. With 13 false positive cases, Cepheid GeneXpert® IV demonstrated the lowest NPA (93.72%) among all three PoC devices. The best analytical accuracy was observed for the Pluslife Mini Dock RHAM-based technology (99.68%), followed by Abbott ID Now™ (99.29%), and Cepheid GeneXpert® IV (95.42%).

One aspect that may reduce test accuracy regardless of RT-PCR or LAMP-based technologies are mutations in the target primer or probe-binding site. SARS-CoV-2 is highly susceptible to mutations in its RNA genome, with different subvariants emerging since its discovery in 2019^36,37^. To further investigate the clinical accuracy of the Pluslife PoC device in identifying SARS-CoV-2 variants, 137 SARS-CoV-2 positive samples with known variants were investigated. With a mean time-to-positive result of 15–20 min, the Pluslife test demonstrated a sound PPA for all SARS-CoV-2 variants, ranging from Delta/Omicron (BA.1) in early 2022 to the current predominant variant XBB.1. There was only one false negative sample amongst the 40 XBB.1 samples analysed, with a relatively low C_T_ value (C_T_ = 31.79). With a PPA of 99.27% (136/137) and an NPA of 100.00% (104/104), the diagnostic accuracy for Pluslife determined by study site 1 was reproduced and confirmed by study site 2, representing two independent clinical evaluation studies.

Although there was no significant difference in the C_T_ values across all SARS-CoV-2 variants (One-way ANOVA, *P* > 0.05), we observed a significant variation in the time-to-positive result across the SARS-CoV-2 variants (*P* < 0.0001). The mean test duration of XBB was significantly higher compared to most other variants (Mann Whitney U test; Delta, *P* < 0.0001; BA.1, *P* = 0.0055; BA.2, *P* = 0.9210; BA.4, *P* = 0.1566; BA.5, *P* = 0.0083), suggesting that the reaction for XBB.1 detection is somewhat slower. The cause of this deviation remains unclear. According to the manufacturer (Pluslife), in-silico analyses confirmed test accuracy, whereby the primers used were aligned to target regions to the different variants of concern (including XBB), suggesting that the test performance of the Pluslife Mini Dock for SARS-CoV-2 is not impaired by any known mutations in the SARS-CoV-2 variants. This observation underlines the importance of post-market surveillance for continued monitoring of clinical accuracy in viral diagnostics.

C_T_ values are a valuable diagnostic tool for monitoring the course of an infection and can be correlated with the viral load, providing a semi-quantitative measure of the degree of infection, early onset of infection in asymptomatic contact persons, and for monitoring recovery progress in severe, hospitalised patients during the SARS-CoV-2 pandemic^7,38,39^. A similar “quantitative measure” quality would be appreciated from a POCT. As the reaction stops when the fluorescence signal exceeds the threshold, a correlation between time-to-result and viral load may exist. Therefore, we investigated whether a correlation existed between the time-to-result of the Pluslife Mini Dock and the C_T_ value. However, no correlation was found (Pearson r = 0.01427, *P* = 0.8695, n = 135). Thus, the RHAM technology only indicated accurately the presence or absence of a viral infection but did not facilitate any significant (semi-) quantitative measurements.

In conclusion, the RHAM technology applied by the Pluslife Mini Dock PoC device demonstrated very good analytical PPA, NPA, and accuracy in identifying SARS-CoV-2 variants compared to RT-PCR results. The major advantage of PoC devices is that special laboratory facilities and trained personnel are not required. This facilitates the decentralised, reliable detection of pathogens of interest directly in a PoC setting. A POCT may be a preferred and better option for many developing countries that lack the necessary infrastructure and facilities. The RHAM technology is an emerging method poised to become of increasing relevance and application in preventing and controlling not only SARS-CoV-2 infections, but also future outbreaks of infectious diseases.

### Supplementary Information

Supplementary Information 1.

Supplementary Information 2.

Supplementary Information 3.

## Data Availability

Original data for the comparative study is archived at Pfützner Science and Health Institute, Mainz, Germany. Data for the variant analysis is stored at DHS—Diagnostic HealthCare Services, Berlin, Germany. The datasets used and/or analysed during the current study are available from the corresponding author upon reasonable request.
