# Supplementary material for: Comparison of the diagnostic accuracy of the Pluslife Mini Dock RHAM technology with Abbott ID Now and Cepheid GenXpert: A retrospective evaluation study
Source: Sci Rep. 2024 Jun 17;14:13978. doi: 10.1038/s41598-024-64406-9 (PMC11183097; doi:10.1038/s41598-024-64406-9)
Supplement: Supplementary file 1 — Supplementary Information 1. [file 41598_2024_64406_MOESM1_ESM.docx]

**Legends for Supplementary data:**

Supplemental data 1: Overview of statistical analysis and raw data of the analysed samples for SARS-CoV-2. Depicted are results using PCR kits from various leading manufacturers compared to the Pluslife Mini Dock. C_T_ values of the samples analysed are also listed. SS_1, study site 1; SS_2, study site 2.

Supplementary data 2: Graphical depiction of the study design and results. Statistical analysis comparing the three point-of-care devices for the detection of SARS-CoV-2 are depicted. Various SARS-CoV-2 variants were identified correctly using the Pluslife Mini Dock.
