# Supplementary figures and images for: Comparison of the diagnostic accuracy of the Pluslife Mini Dock RHAM technology with Abbott ID Now and Cepheid GenXpert: A retrospective evaluation study
Source: Sci Rep. 2024 Jun 17;14:13978. doi: 10.1038/s41598-024-64406-9 (PMC11183097; doi:10.1038/s41598-024-64406-9)

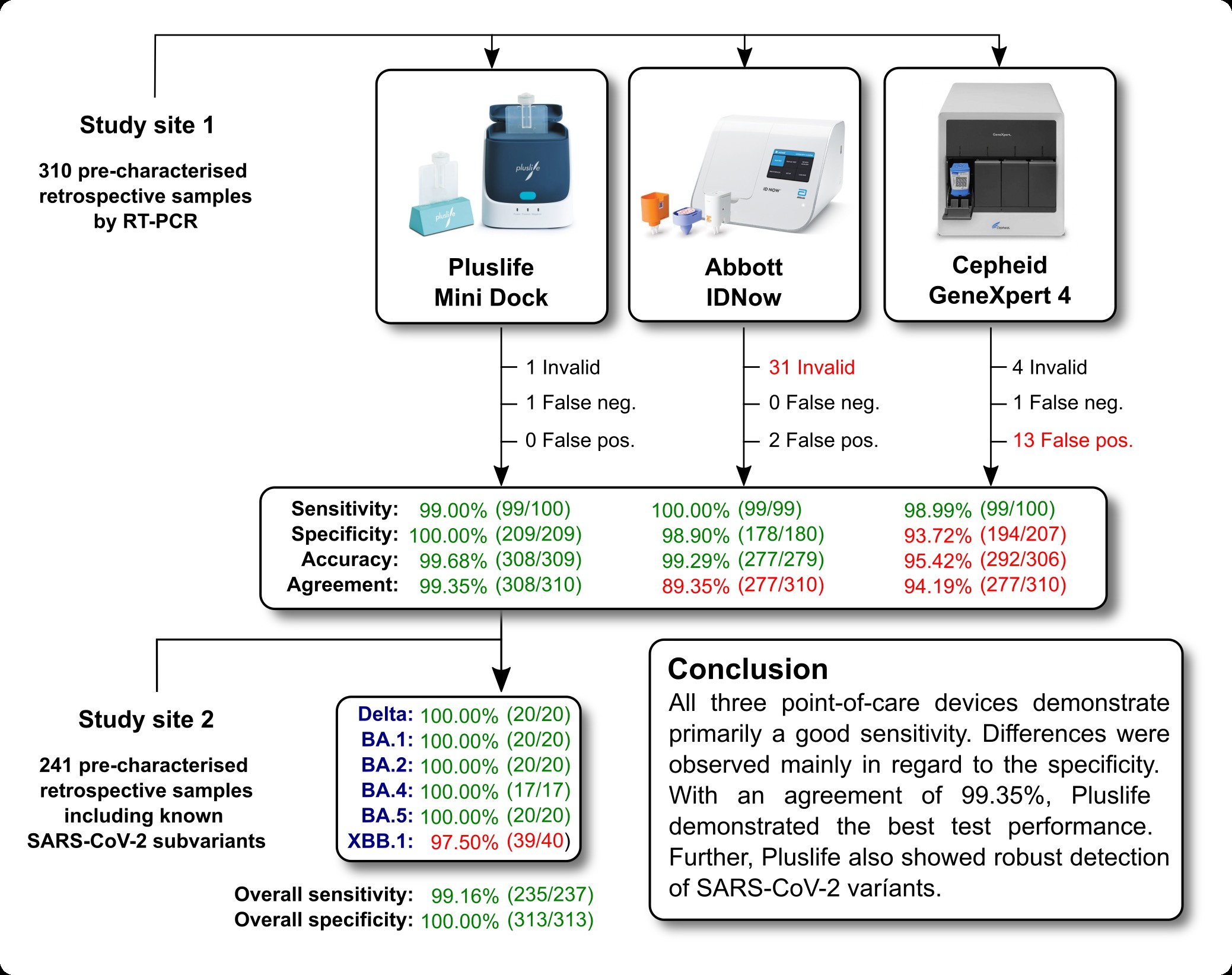

Supplement: Supplementary file 3 — Supplementary Information 3. [file 41598_2024_64406_MOESM3_ESM.jpg]
